# Co-construct, implement and evaluate an intervention to prevent a sedentary lifestyle in children

**DOI:** 10.1093/eurpub/ckac131.330

**Published:** 2022-10-25

**Authors:** S Laujac, M Cholley-Gomez, J Franceschi, A Rozand, L Pallier, JC Basson, A Vuillemin, P Duché, C Delpierre, M Carayol

**Affiliations:** 1 IAPS Laboratory, University of Toulon, Toulon-La Garde, France; 2 Comity of Health Education of Var Department, CODES 83, Toulon-La Garde, France; 3 CRESCO Laboratory - EA 7419, University of Toulouse III Paul Sabatier, Toulouse, France; 4 IFERISS FED 4142, University of Toulouse, Toulouse, France; 5 LAMHESS, Côte d’Azur University, Nice, France; 6 CERPOP UMR 1295, National Institute of Health and Medical Research, Toulouse, France

## Abstract

**Background:**

In children a sedentary lifestyle is associated with the development of chronic diseases, as well as unfavorable body composition and physical condition, lower levels of self-esteem, sociability, and school results. In Europe 39,8% of children (6-9 years) spend on average more than 2h/day in front of a screen, and 14,6% over 3h/day. In this context, designing and testing effective interventions to decrease sedentary behavior in children is a major public health research gap. The CIPRES intervention aims to reduce sedentary time in school-age children (7-10 years).

**Methods:**

The CIPRES intervention is co-constructed with key local actors, by using a socio-ecological approach, and theory-based on the transcontextual model. The intervention is evaluated by a cluster-randomized controlled study currently ongoing. The target population is made up of 1000 children from 13 primary schools (in years 4-5) from southeast of France with different levels of social deprivation. Main outcomes are assessed by accelerometer and questionnaires before (T0) and after a six-week intervention (T1) and include sedentary behavior, physical activity (PA) and variables of the transcontextual model.

**Results:**

Preliminary data were available in 152 children (53 intervention and 99 control). There was no significant difference across the time in sedentary time between intervention and control group (p = 0.11; η^2^=0,017); however, moderate-to-vigorous physical activity (MVPA) tended to be better in intervention vs control (p = 0.06; η^2^=0,023). In the intervention group, sedentary time and MVPA were significantly better across the time (p = 0.028 and p = 0.011, respectively) for children having a father with a university diploma as compared to children having a father with a lower education level.

**Conclusions:**

Preliminary results on a small group of subjects (n = 152) are encouraging and suggest a positive impact of the intervention on children. More results will be available by summer 2022.

**Key messages:**

• Based on the socio-ecological model, the CIPRES intervention aims to prevent sedentary in school-aged children.

• Preliminary data suggests a favorable impact of the intervention on physical activity.

